# Raman-Based Diagnostics of Stalk Rot Disease of Maize Caused by *Colletotrichum graminicola*

**DOI:** 10.3389/fpls.2021.722898

**Published:** 2021-08-16

**Authors:** Charles Farber, John S. Bennett, Tianyi Dou, Yousef Abugalyon, Dillon Humpal, Lee Sanchez, Katie Toomey, Michael Kolomiets, Dmitry Kurouski

**Affiliations:** ^1^Department of Biochemistry and Biophysics, Texas A&M University, College Station, TX, United States; ^2^Department of Plant Pathology and Microbiology, Texas A&M University, College Station, TX, United States; ^3^The Institute for Quantum Science and Engineering, Texas A&M University, College Station, TX, United States; ^4^Department of Molecular and Environmental Plant Science, Texas A&M University, College Station, TX, United States

**Keywords:** anthracnose stalk rot, *Colletotrichum graminicola*, disease diagnostics, maize, Raman spectroscopy, Zea mays

## Abstract

Stalk rot caused by *Colletotrichum graminicola* is a disease of worldwide importance. Stalk rot is difficult to detect at the early stages of infection because the fungus colonizes the tissues inside the maize stem. Current diagnostic methods are time-consuming, laborious, and destructive to the stem tissue. We utilized Raman spectroscopy to follow the development of stalk rot in three different maize genotypes grown either in the field or the greenhouse. We then used the acquired spectra to calibrate statistical models to differentiate amongst the different disease timepoints and the genotypes themselves. This non-invasive spectroscopic method enabled high-accuracy identification of stalk rot based on both stalk and leaf spectra. We additionally found that leaf spectra were favorable for identifying maize by genotype. Finally, we identified Raman bands that showed correlation with the sizes of stalk rot-associated lesions in the stems. We demonstrated that Raman spectroscopy is a viable tool for detection of stalk rot disease, as well as potent for the differentiation of maize genotypes.

## Introduction

*Colletotrichum graminicola* is one of the most economically disruptive fungi of corn and the causative agent of a disease associated with 5–20% corn yield loss in the United States alone (Sukno et al., 2008, 2014; Jackson-Ziems et al., 2014). This pathogen can infect and cause disease primarily the leaves and stem of maize, where it leads to anthracnose leaf blight (ALB) and stalk rot diseases, respectively. These are associated with development of sunken necrotic lesions starting 48–72 h after initial infection (O’Connell et al., 1985). Resistance to anthracnose stalk rot (ASR) is correlated with the production of specific plant hormones and metabolites. Specifically, Gorman and coworkers found that reduced jasmonic acid (JA) and increased salicylic acid (SA) levels measured in JA-deficient mutants of maize were associated with increased resistance to *C. graminicola* in ASR (Gorman et al., 2020). SA is produced from compounds such as phenylalanine and cinnamic acid. These are examples of phenylpropanoids, a family of organic compounds comprising many essential components for defense against herbivores and pathogens. Furthermore, Vargas et al. (2012) demonstrated that anthocyanins, hydroxycinnamic acid derivatives, and peroxides were produced in response to ALB. As key defense molecules such as phenylpropanoids can be assigned to specific vibrational bands in Raman spectra, Raman spectroscopy (RS) may prove ideal for detection of diverse biochemical changes involved in plant defense against diseases such as ASR.

Confirmatory diagnosis of stalk rot can be used to guide application of fungicides allowing for efficient disease control and maximization of crop yield (Farber et al., 2019a). There are several molecular and imaging techniques that can be used to detect stalk rot diseases (Raza et al., 2015). For instance, polymerase chain reaction (PCR) and enzyme-linked immunosorbent assay (ELISA) are commonly used for confirmatory diagnostics of fungal diseases (Alvarez and Lou, 1985; Li et al., 2006; Lievens et al., 2006). However, these molecular methods have their own limitations. ELISA, for instance, including double and triple antibody ELISA, has low sensitivity, photobleaching instability and poor specificity to related pathogen strains (Lievens et al., 2006; Sankaran et al., 2010). PCR has limited portability, high labor and cost requirements, need of specific expertise, difficulty in screening an entire field, and is essentially destructive to the analyzed specimen (Wallner et al., 1993; Chitarra and Bulk, 2003; Sankaran et al., 2010). Recently, several imaging techniques, such as red–green–blue (RGB) and hyperspectral imaging, as well as thermography, have been proposed as alternative non-invasive approaches to molecular methods of analysis. These techniques allow for fast imaging of broad field areas (Baena et al., 2017). However, they suffer from poor specificity since the stress detection is based on changes in plant color or temperature. Such changes could also be caused by a variety of factors such as seasonal variations in temperature, nutrient deficiencies, or unrelated diseases. Additionally, despite quick imaging, data analysis typically requires several weeks before conclusions can be drawn (Mahlein et al., 2012). Accounting for the fast growth of plants and rates of development of biotic and abiotic stresses, such slow data analysis turnaround drastically decreases practical applications of RGB, hyperspectral imaging, thermography, and other imaging techniques (Farber et al., 2019a).

Raman spectroscopy is a label-free, non-invasive, non-destructive spectroscopic technique that provides information about the chemical structure of analyzed specimens (Cardona, 1975). The Raman effect is based on inelastic scattering of photons by molecules that are being excited to higher vibrational or rotational states (Kurouski et al., 2015). RS is commonly used in food chemistry (Almeida et al., 2010), electrochemistry (Zeng et al., 2016), forensics (Virkler and Lednev, 2009; López-López et al., 2013) and materials science (Cantarero, 2015). It is capable of monitoring changes in protein secondary structure (Kurouski et al., 2012), elucidation of composition and origin of body fluids (Virkler and Lednev, 2009) as well as gun-shot residues (Bueno and Lednev, 2013). Although RS is generally known to be a laboratory-based technique, the past decade has seen several developments of portable Raman spectrometers, which has enabled the utilization of RS directly in the field (Yeturu et al., 2016; Sanchez et al., 2019a,b, 2020a). This technological development sparked the interest of agronomists, plant pathologists, and plant biologists in utilization of this technology for analysis of the plant health status.

Our group discovered that (RS) can be used for non-invasive, non-destructive, fast, chemical-free, and confirmatory diagnostics of plant diseases (Farber et al., 2019a). We showed that using RS, fungal diseases of maize, wheat, and sorghum can be diagnosed with nearly 100% accuracy (Egging et al., 2018). RS can also be used for detection of viral diseases of wheat and rose, as well as the bacteria that cause Huanglongbing (HLB or Citrus Greening) in citrus trees (Farber et al., 2019b; Sanchez et al., 2019a,b). This diagnostic approach is based on detection of pathogen-induced changes in structure and composition of plant molecules. Such changes are unique for each pathogenic species. For example, our group found that the Raman spectra of maize kernels infected with different fungi varied dramatically (Farber and Kurouski, 2018). Thus, RS has species-level sensitivity in pathogen diagnostics. Moreover, we have discovered that RS can be used to predict abiotic stresses associated with nutrient deficiencies of citrus trees (Sanchez et al., 2019b). Lastly, we showed that RS can be used for the accurate identification of maize and peanut varieties based on spectroscopic signatures of their leaves and seeds. The accuracy of such non-invasive plant phenotyping ranges from 80 to 95% (Krimmer et al., 2019; Farber et al., 2020). These results demonstrate that RS can be used in field and greenhouses settings for rapid phenotyping of plants including prediction of nematode-resistance and nematode-susceptibility of analyzed varieties. Moreover, RS allows for non-invasive and non-destructive assessment of nutrient content of seeds providing information about their carbohydrate, protein, fiber, as well as oils and unsaturated fatty acids in peanut seeds (Krimmer et al., 2019; Farber et al., 2020). These findings suggest that RS can be used for both diagnostics of plant stresses and digital phenotyping. This is critically important to enable digital sorting of seeds with simultaneous prediction of their economic/nutrient values. One can envision that RS can be used to assist breeding of plants by revealing necessary qualities of germplasm much faster than molecular methods of analyses.

Expanding upon these findings, in this study we investigated the accuracy of RS in the identification of ASR disease caused by *C. graminicola* in both greenhouse- and field-grown maize. We collected spectra from both leaves and stalks of three maize varieties that contrast in their resistance to ASR (MP305 (resistant), *lox4-7* (susceptible), and B73 (intermediately susceptible) at day 4 (D4), 8 (D8), and 12 (D12) post-inoculation. We also investigated whether RS could be used to identify different maize varieties to determine whether Raman-based stalk rot diagnostics should be developed in a variety-specific approach. Lastly, we performed quantification of lesions and correlated these data with spectroscopic signatures of leaves and stalks of plants.

## Materials and Methods

### Plant and Fungal Materials

As previously described, the mutant allele of *lox4-7* was identified by PCR screening for the *Mutator*-transposon insertional resource at DuPont-Pioneer, Inc. (currently, Corteva) for insertions in this gene (Park, 2012; Christensen et al., 2013). The *lox4-7* allele is a confirmed exon-insertional mutant backcrossed to BC7 stages in the B73 genetic background and confirmed by PCR for homozygous mutant status. The maize inbred MP305 was obtained from Dr. Paul Williams (Mississippi State University). Seeds of these genotypes were planted with 4–6 seeds per pot in Metro Mix 360 RSi soil (Sun Gro Horticulture). Seedlings were thinned to two seeds per pot within the next 2 weeks. Plants were watered every 3–4 days and 20 g of Osmocote Blend 19-5-9 slow-release fertilizer (Everris NA Inc.) were applied to each pot at about 2 and 6 weeks after planting. For the field trial, seeds were sown in plots of 25 seeds per plot with 4 reps per genotype in a randomized complete block design. *Colletotrichum graminicola* (1.001 strain) was cultured from stock plates from the lab of Dr. Young-Ki Jo (Texas A&M University) on full strength potato dextrose agar for at least 14 days at 23–25°C. Spore suspensions were prepared as previously described (Gao et al., 2007).

### Infection of Stalks With *C. graminicola*

The point at which 50% of the plants of each genotype silked (mid-silking) was determined and leaves above the ear and surfaces of 2nd and 4th internodes underneath the leaf sheath of plants were scanned with a handheld Raman spectrometer 10–13 days after mid-silking. The first viable (not dried out) leaf above the 4th internode was also scanned at these same timepoints. Ten plants per genotype were inoculated following previously described methods (Thon et al., 2002; Gao et al., 2007). Briefly, the bottom four internodes above the last node with brace roots were wounded with an 18G hypodermic needle inserted to 1/4 inch depth. Sterile cotton swabs were used to apply either control treatment (0.01% Tween in sterile distilled water) or a spore suspension of 1 × 10^6^ spores/mL of *C. graminicola* and wrapped in place on the wound site with parafilm to create a humid chamber. Infections were allowed to progress to selected time points of 4, 8, and 12 dpi. At each data collection, 10 plants were randomly selected from each genotype × treatment combination to be scanned at the four most basal internodes above the brace roots and harvested to be split and photographed. Proportion or percent infection was determined by measuring lesion area in addition to internode area and dividing lesion area by internode area. This accounts for the possibility of smaller internodes being completely colonized and the fungus thus not having more tissue to infect. The ratios of infected areas were compared to each other using JMP and subject to two-way analysis of variance (ANOVA) to test for interaction and main fixed effects of the genotype and days past inoculation. In both greenhouse and field trials where the genotype × dpi interaction was significant, genotype means were compared by Tukey’s HSD test at α = 0.05 at each dpi separately.

### Raman Spectroscopy

Raman spectra were collected with a hand-held Resolve Agilent spectrometer equipped with an 830-nm laser source. The following experimental parameters were used for all collected spectra: 1 s integration time, 495 mW power, and baseline spectral subtraction by device software. Previously reported experimental results demonstrated absence of photodegradation of plant material at these experimental conditions. We also observed neither visual signs of laser-induced photodegradation of rice leaves during spectral acquisition nor any noticeable structural changes in plants in the control group of plants (Sanchez et al., 2020b). Stalk scans were acquired while gently pressing the device onto a flat side of the stalk to maximize the contact between the device and stalk. This contact guarantees that the stalk will be at the focal point of the laser and therefore generate the most signal. Leaf scans were acquired by pressing the instrument into the leaves in a similar way. These leaf scans were taken from the first viable (not dead or dried out) leaf above the 4th internode from points near the center, but not along the central vein. At each timepoint, five plants from each treatment group were selected for analysis. 10 spectra were acquired from the stalks and six from the leaves of each plant. Control plants were retained while treated plants were harvested for lesion analysis.

### Spectral Data Analysis

PLS_Toolbox (Eigenvector Research Inc.) was used for statistical analyses of the collected Raman spectra. Before multivariate analysis, spectra were preprocessed with combinations of the following preprocessing steps to build the best model, as informed by the model optimizer: area normalization, standard normal variate scaling, mean centering, and first derivative. Partial least squares discriminant analysis (PLS-DA) was performed to determine the number of significant components and identify spectral regions that best explained separation between the classes. All models described in the paper except the genotype identification models (Tables 1, 2) are binary models constructed with spectra from mock-inoculated control plants scanned the same day as the corresponding infected plants. “Ctrl+D4 v D12” models are described in more detail in the “Results and Discussion” section. All reported accuracy values are the averages of the cross-validation true positive rate for all classes in the model.

**TABLE 1:** Accuracy of leaf-based identification of MP305, *lox4-7*, and B73 in the greenhouse and field.

**Genotype**	**Greenhouse (%)**	**Field (%)**
MP305	82	77
*lox4-7*	55	90
B73	83	81
Average	73	82

**TABLE 2:** Accuracy of stalk-based identification of MP305, *lox4–7*, and B73 in the greenhouse and field.

**Genotype**	**Greenhouse (%)**	**Field (%)**
MP305	100	100
*lox4-7*	89	73
B73	89	71
Average	92	81

### Correlation Analysis

Correlations between averaged lesion size per internode scanned and the associated Raman spectra of those internodes were conducted using MATLAB. The MATLAB command corrcoef was used to obtain the *r* and *p* values for each correlation.

## Results and Discussion

### Plant Analysis

**FIGURE 1:**
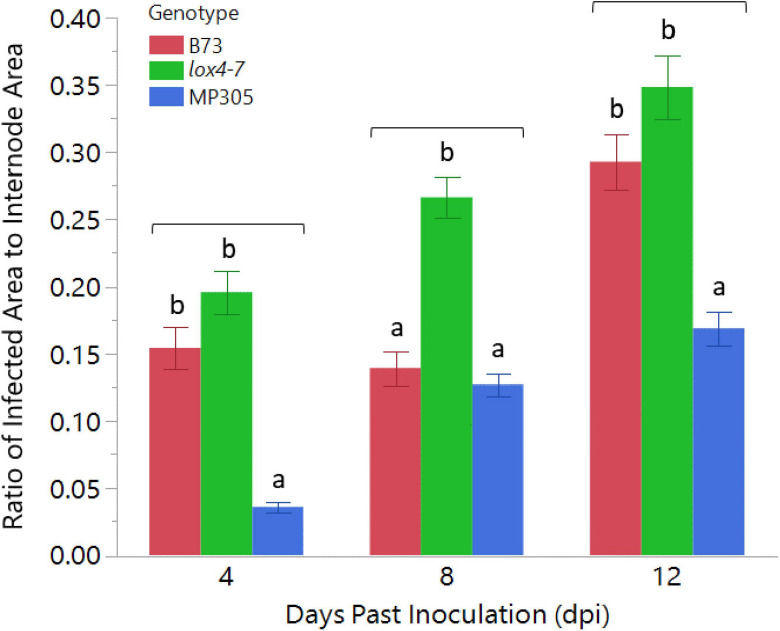
Anthracnose stalk rot progression in wild type B73, *lox4-7*, and MP305 maize in greenhouse trial. *lox4-7* was significantly more susceptible, as expected, than WT B73 at 8 and 12 dpi, and MP305 displayed significant resistance to lesion development at all time points. Bars indicate ratios of surface areas of *Colletotrichum graminicola*-infected lesions to areas of each respective internode at 4-, 8-, and 12-days post inoculation (Mean ± SE; different letters indicate significant differences between means within the same time point, determined by Tukey’s HSD, *p* < 0.05).

To investigate the ability of RS to differentiate between maize varieties that contrast in their resistance or susceptibility levels to ASR, we inoculated stalks of the susceptible *lox4-7* mutant, its near-isogenic wild type recurrent parent, (NIL-WT) B73 inbred, which displays intermediate susceptibility and MP305 inbred with *C. graminicola*. Maize *lox4* mutants have previously been shown to be significantly more susceptible to ASR due to substantially lower levels of SA, the major defense hormone against this hemibiotrophic pathogen (Damarwinasis, 2018). Two-way ANOVA indicated there was a significant effect in genotype (*p* < 0.0001) as well as in dpi (*p* < 0.0001). As ANOVA additionally revealed a significant effect in genotype × dpi (*p* = 0.0157), genotype means were only compared to each other by Tukey’s HSD within the same time point. MP305 displayed expected resistance to the pathogen as described previously, showing a ratio of infected area of only 0.17 compared to 0.35 in *lox4-7* and 0.29 for B73 (Figure 1; Jung et al., 1994).

**FIGURE 2:**
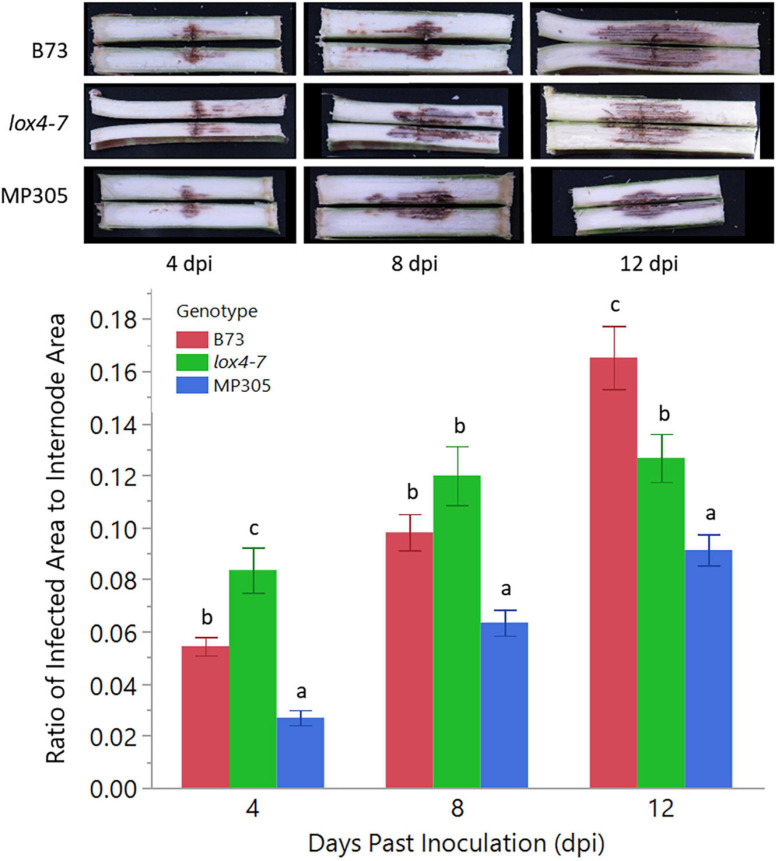
Anthracnose stalk rot (ASR) disease progression in the three genotypes under field conditions. *lox4-7* mutant showed a significantly more rapid development of stalk rot at early time points compared to WT B73, although by 12 dpi, WT showed greater disease progression than *lox4-7*. MP305 was again significantly resistant at each time point. Bars indicate the average ratios of areas of *C. graminicola*-infected lesions to surface areas of each respective internode (cm^2^/cm^2^) in wild type B73 inbred, *lox4-7* mutant, and MP305 inbred maize at 4-, 8-, and 12-days post inoculation (±SE; different letters indicate significant differences between means within the same time point, determined by Tukey’s HSD, *p* < 0.05).

To determine if this technique would perform equally under field conditions, we planted B73, *lox4-7* mutant, and MP305 in a randomized complete block design in the field and inoculated stalks with *C. graminicola* when plants were 10–14 days past mid-silking. Two-way ANOVA indicated there was a significant effect in genotype (*p* < 0.0001) as well as in dpi (*p* < 0.0001). ANOVA again revealed a significant effect in genotype × dpi (*p* = 0.0002) so genotype means were only compared to each other by Tukey’s HSD within the same time point. MP305 was consistently the most resistant at all three time points, while the susceptible mutant *lox4-7* initially showed significantly more rapid disease progression compared to WT B73 at 4 dpi. By 12 dpi, however, B73 unexpectedly had significantly more infected area compared to *lox4-7* and MP305, most likely due to plant-to-plant variation (Figure 2).

### Spectroscopic Analysis

**FIGURE 3:**
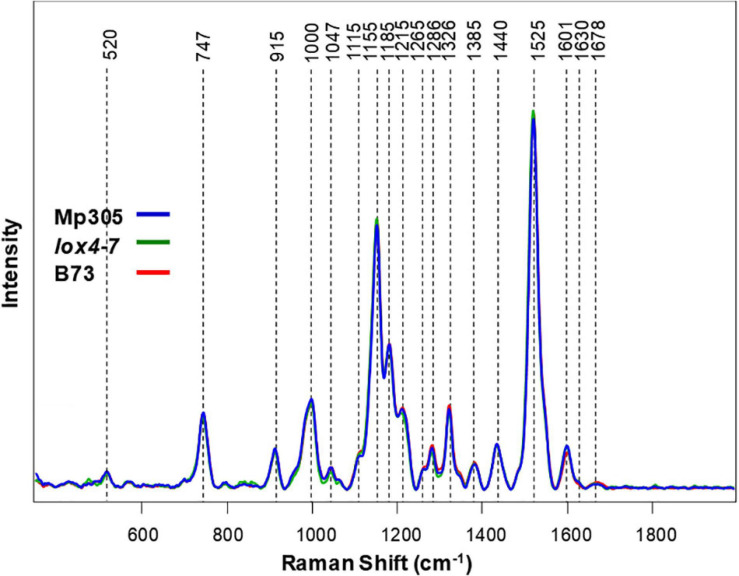
Raman spectra collected from leaves of non-inoculated control plants from all three maize varieties.

Raman spectra collected from leaves of all three corn varieties exhibited vibrational bands that could be assigned to pectin (747 cm^–1^), cellulose (520, 915, 1,047, and 1,115 cm^–1^), carotenoids (1,000, 1,155, 1,185, and 1,525 cm^–1^), phenylpropanoids (1,601–1,630 cm^–1^), protein (1,678 cm^–1^), and aliphatic vibrations (1,215, 1,286, 1,326, 1,385, and 1,440 cm^–1^) (Figure 3 and Table 3).

**TABLE 3:** Spectral band assignments for leaves and stalks of corn.

**Band**	**Vibrational mode**	**Assignment**
375	Associated with cellulose crystallinity	Cellulose (Agarwal et al., 2010)
520	*ν*(C–O–C) Glycosidic	Cellulose (Edwards et al., 1997)
640	δ(C–C)	Lignin (Larsen and Barsberg, 2010)
742–747	γ(C–O–H) of COOH	Pectin (Synytsya et al., 2003)
804	δ ring vibration	Terpenes (Schulz et al., 2005b)
900–918	*ν*(C–O–C) In plane, symmetric	Cellulose, lignin (Edwards et al., 1997)
987	*ν*(CO)_ring_, *ν*(CC)_ring_, and β(CCH)	Carbohydrates (Wiercigroch et al., 2017)
1,000	In-plane CH_3_ rocking of polyene aromatic ring of phenylalanine	Carotenoids (Schulz et al., 2005a); protein
1,040–1,047	*ν*(C–O) + *ν*(C–C) + δ(C–O–H)	Cellulose, lignin (Edwards et al., 1997)
1,093	*ν*(C-O) + *ν*(C-C) + δ(C-O-H)	Carbohydrates (Almeida et al., 2010)
1,115	Sym *ν*(C–O–C), C–O–H bending	Cellulose (Edwards et al., 1997)
1,121	*ν*(C–O) + *ν*(C–C) + δ(C–O–H)	Carbohydrates (Almeida et al., 2010)
1,155	C–C Stretching; v(C–O–C), v(C–C) in glycosidic linkages, asymmetric ring breathing	Carotenoids (Schulz et al., 2005a), carbohydrates (Wiercigroch et al., 2017)
1,170	C–OH	Lignin (Blaschek et al., 2020)
1,185–1,186	*ν*(C–O–H) Next to aromatic ring+σ(CH)	Lignin (Mary et al., 2012; Agarwal, 2014)
1,202	Aromatic ring modes of phenylalanine and tyrosine	Proteins (Zheng et al., 2004)
1,215	δ(C-C-H)	Aliphatics (Yu et al., 2007), xylan (Agarwal, 2014)
1,265–1,267	Guaiacyl ring breathing, C–O stretching (aromatic); -C=C–	Lignin (Cao et al., 2006), unsaturated fatty acids (Jamieson et al., 2018)
1,286	δ(C–C–H)	Aliphatics (Yu et al., 2007)
1,326	δCH_2_ bending	Aliphatics, cellulose, and lignin (Edwards et al., 1997)
1,335	δ(CH_2_) + δ(CH_3_)	Aliphatics (Yu et al., 2007)
1,385	δCH_2_ bending	Aliphatics (Yu et al., 2007)
1,424–1,460	δ(CH_2_) + δ(CH_3_)	Aliphatics (Yu et al., 2007)
1,525	–C=C– (in plane)	Carotenoids (Adar, 2017; Devitt et al., 2018)
1,601–1,627	*ν*(C–C) Aromatic ring + σ(CH)	Lignin (Agarwal, 2006; Kang et al., 2016)
1678	C=O Stretching, amide I	Proteins (β-sheet) (Devitt et al., 2018)
1698	COOH	Carboxylic acids

**FIGURE 4:**
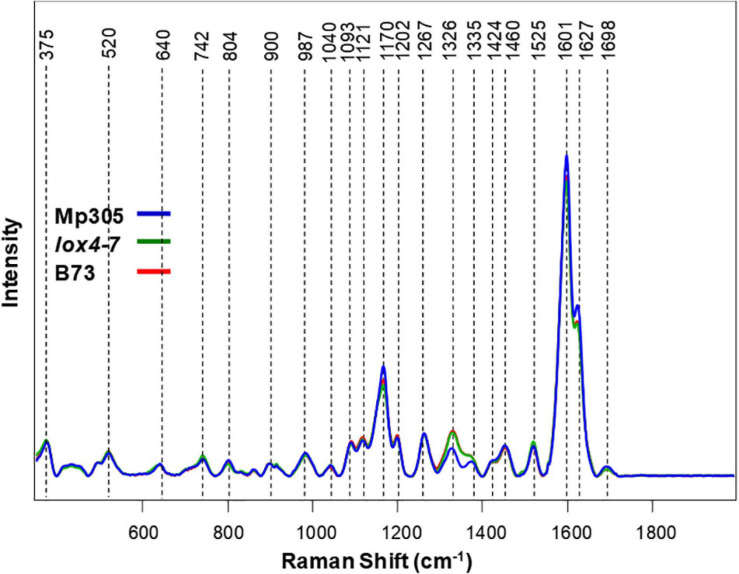
Raman spectra collected from stalks of non-inoculated control plants from all three maize varieties.

Spectroscopic analysis of plant stalks revealed that vibrational signatures of phenylpropanoids (1,601–1,627 cm^–1^) dominate the spectra (Figure 4). We also observed vibrational bands that can be assigned to pectin (742 cm^–1^), cellulose (520, 915, 1,040, 1,093, and 1,121 cm^–1^), carotenoids (1,525 cm^–1^), carboxylic acids (1,698 cm^–1^), and aliphatic vibrations (1,326, 1,335, 1,424, and 1,460 cm^–1^) (Figure 4 and Table 3). These results point to substantial differences in the structure of leaves and stalks. Next, we constructed PLS-DA models using the pre-inoculation spectra to investigate whether RS can be used to identify these plant genotypes based on their spectroscopic signatures.

Our results showed that based on the spectroscopic signatures of leaves, MP305, *lox4-7*, and B73 could be differentiated from each other with average accuracies of 73 and 82% in the greenhouse and field, respectively (Table 1). For instance, *lox4-7* and B73 could be correctly identified in the greenhouse with 55 and 83% accuracy, respectively. In the models based on field-collected data, the accuracy of identification of these two genotypes was 90 and 81%, respectively. Since these two genotypes are about 99.7% identical at the genome level, being back-crosses at the seventh stage, such a high accuracy of their identification reflects truly remarkable sensitivity of RS.

Utilization of spectroscopic signatures of stalks provides more accurate identification of maize varieties in the greenhouse; however, nearly identical accuracy of identification was obtained in the field. Specifically, the three genotypes could be differentiated with average accuracies of 92 and 81% in the greenhouse and field, respectively (Table 2). We also observed substantially higher accuracy of differentiation between *lox4-7* and B73 in the greenhouse with 89% accuracy. In the field, the accuracy of identification of these two genotypes was 73 and 71%, respectively. Based on these results, we can conclude that spectral readings from stalks are better suited for identification of plant varieties in the greenhouse, whereas both leaves and stalks can be used for high accuracy of variety identification in the field. These findings suggest that differences between spectroscopic signatures of maize varieties likely originated from inherent differences in their metabolism. These results confirm our previously reported findings that highlighted high sensitivity of RS to inherent plant metabolism, which is different between plant species and their varieties. These results also suggest that Raman-based approach in stalk rot diagnostics likely will require the development of variety-specific chemometric models.

### Diagnostics of Stalk Rot in Greenhouse and Field

#### Leaves

Considering inherent differences between MP305, *lox4-7*, and B73, we developed chemometric models for each genotype that enabled accurate prediction of stalk rot based on spectra collected from leaves of plants grown in the greenhouse. We found that at D4 (pre-symptomatic stage), RS enabled 88% accurate identification of stalk rot in B73, 84 and 61% accurate disease prediction in *lox4-7* and MP305, respectively (Table 4). At the middle stage of infection (D8), the accuracy of stalk rot identification slightly decreased for B73 and *lox4-7* and increased for MP305. At the same time, at the late stage (symptomatic plants), the accuracy of prediction was found to be 90% for B73 and *lox4-7*, and 61% on MP305. These results suggest that changes in plant biochemistry associated with the infection are less prominent in MP305 compared to B73 and *lox4-7*. This results in lower accuracy of disease diagnostics in MP305.

**TABLE 4:** True positive rates of identifying control and treated plants based on Raman spectra of leaves from greenhouse-grown maize.

**Genotype**	**B73**		***Lox4-7***		**MP305**	
**Treatment**	**Ctrl (%)**	**Treat (%)**	**Ctrl (%)**	**Treat (%)**	**Ctrl (%)**	**Treat (%)**
D4	95	82	85	84	55	68
D8	71	70	79	74	83	60
D12	95	86	100	80	68	55

While the scope of this study did not include metabolome profiling to shed light into the biochemical changes that may explain the difference between B73 and MP305, we speculate that the resistant MP305 responded more strongly and early to infection that may have led hypersensitive response-like reaction due to the existence of a major disease resistance gene, Rcg1, in this germplasm and thus strong biochemical changes but only at the early time points after infection (Frey et al., 2011). Regardless, our results suggest that changes in the plant biochemistry associated with the stalk rot are stronger at early stages of the disease development (D4). This can be explained by activation of defense mechanisms of the plant, which results in substantial changes in plant metabolism at the early stages. Consequently, this allows for highly accurate pre-symptomatic Raman-based diagnostics of stalk rot diseases in maize. Our results also suggest that intensity of changes in the plant metabolism decreases at D8. Lastly, high accuracy of disease diagnostics at late stages (D12) suggests that drastic changes in the plant metabolism occur upon substantial progression of stalk rot, which ultimately lead to the death of the plant tissue. These findings demonstrate that accuracy of stalk rot disease diagnostics in maize is 1) variety-specific and 2) time-specific. Similar results have been obtained for field-grown MP305, *lox4-7*, and B73 (Table 5).

**TABLE 5:** True positive rates of identifying control or treated plants based on Raman spectra of leaves from field-grown maize.

**Genotype**	**B73**		***Lox4-7***		**MP305**	
**Treatment**	**Ctrl (%)**	**Treat (%)**	**Ctrl (%)**	**Treat (%)**	**Ctrl (%)**	**Treat (%)**
D4	83	76	65	53	45	55
D8	51	66	72	60	59	62
D12	89	23	70	65	62	60
Ctrl/D4 v D12*	86	88	74	52	79	59

*Ctrl is Ctrl/D4; D12 is Treat.

As in the greenhouse-based experiment, MP305 exhibited lower accuracy of diagnostics of stalk rot in the field than B73 and *lox4-7* at all stages of disease development (Table 5). We also found substantially lower accuracy of stalk rot diagnostics at D4, D8, and D12 compared to the corresponding accuracies observed in the greenhouse experiment. These results suggest that other stresses that plants experience while in the field may influence the prediction performance. These other stresses lower the accuracy of determination of one specific (stalk rot) stress in maize. Nevertheless, our results show that infection of B73 can be detected with 79% accuracy on the early stage (D4), whereas the accuracy of correct identification of stalk rot for *lox4-7* and MP305 were 59 and 50%, respectively. The accuracy of stalk rot diagnostics slightly increased at D12 for both *lox4-*7 and MP305 and became 67 and 61%, respectively. At the same time, it decreased to 56% for B73.

We also found that an alternative approach can be used to substantially improve the accuracy of stalk rot diagnosis in the field. Instead of direct comparison of spectra collected from control and infected plants at a single time point, we propose to use D4 and D12 spectra, both control and infected, to predict occurrence of stalk rot. This approach mitigates potential issues from comparing plants across time points, where changes in the plants could be due to either disease progression or simply changes associated with growth. The idea is that we want to simplify the system to determining whether plants are in the early stages/uninfected or are in the later stages of infection. Our findings show that if signatures of late (D12) or early-type (Control and D4) infected plants are used to build PLS-DA models, highly accurate diagnostics of early (D4) and late (D12) stalk rot disease can be performed. Such models are based on the identification of the relative degree of disease progression in plants. We found that this approach enabled 69% accurate disease identification in MP305, 63% accurate identification in *lox4-7*, and 87% accurate identification in B73 (Table 5).

#### Stalks

We also explored the accuracy of stalk rot diagnostics by Raman-based analysis of maize stalks. Our results show that at the early stages (D4), spectroscopic analysis of stalks provides higher accuracy of Raman-based diagnostics of stalk rot. Specifically, we found that the disease can be correctly predicted with 69% accuracy in MP305, 92% in *lox4-7*, and 93% in B73 (Table 6). This can be explained by proximity of the pathogen to the stalk tissue. As previously observed in the analysis of the leaves, we found that the accuracy of diagnostics decreased for all three genotypes at D8. This suggests that although proximity to the diseased tissue can increase the accuracy of disease detection, stalk rot likely triggers plant systemic responses. At D12, accuracy of stalk rot was found to be 71% for MP305, 98% for *lox4-7*, and 89% for B73. Based on these results, one can conclude that in terms of accuracy of prediction, analysis of stalks is more advantageous compared to the analysis of leaves for detection of ASR on *lox4-7* and MP305 and less advantageous for B73. These results also demonstrate that biochemical changes due to stalk rot detected by RS are stronger in stalks than in leaves. Additionally, the magnitude of changes in biochemical profiles is time- and variety-dependent.

**TABLE 6:** True positive rates of identifying control and treated plants based on Raman spectra of stalks from greenhouse-grown maize.

**Genotype**	**B73**		***Lox4-7***		**MP305**	
**Treatment**	**Ctrl (%)**	**Treat (%)**	**Ctrl (%)**	**Treat (%)**	**Ctrl (%)**	**Treat (%)**
D4	98	89	92	93	65	73
D8	92	85	88	88	86	48
D12	97	82	98	98	56	89

Accuracy of stalk rot diagnostics was found to be much lower in the field experiment. We found that at D4, the disease can be correctly predicted in MP305 with 57% accuracy, in *lox4-7* with 63% accuracy, and for B73 with 76% accuracy (Table 7). These results confirm previously discussed findings that accuracy of pre-symptomatic stalk rot diagnostics is variety- and growth conditions-specific. We observed a decrease in the accuracy for stalk-based detection of the infection. Although detection of symptomatic stage of stalk rot is low (D12), we found that the approach described above for relative prediction of disease progression using D4 and D12 spectra enables highly accurate diagnosis of this disease in the field. Specifically, the model that utilizes D4/D12 spectra, allows for detection of stalk rot in the field with 82% for B73, 69% for MP305, and 71% for *lox4-7* (Table 7).

**TABLE 7:** True positive rates of identifying control and treated plants based on Raman spectra of stalks from field-grown maize.

**Genotype**	**B73**		***Lox4-7***		**MP305**	
**Treatment**	**Ctrl (%)**	**Treat (%)**	**Ctrl (%)**	**Treat (%)**	**Ctrl (%)**	**Treat (%)**
D4	72	81	69	58	69	46
D8	65	62	68	52	63	52
D12	91	86	55	72	54	58
Ctrl/D4 v D12*	87	77	63	79	69	70

*Ctrl is Ctrl/D4; D12 is Treat.

### Digital Selection of Maize Varieties Enabled by Raman-Based Prediction of Stalk Rot Resistance

**FIGURE 5:**
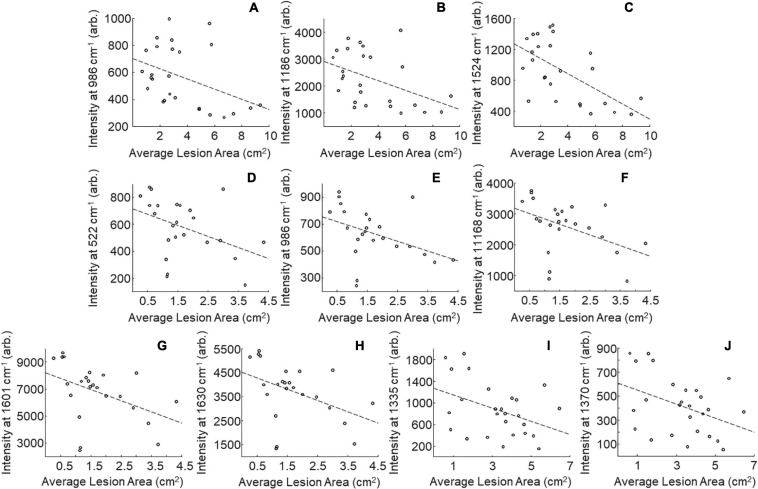
Scatter plots of intensity at selected peaks versus averaged lesion size for field-grown plants. B73: 986 **(A)**, 1,168 **(B)**, and 1,524 **(C)** cm^–1^; MP305: 522 **(D)**, 986 **(E)**, 1,168 **(F)**, 1,601 **(G)**, and 1,630 **(H)**; *lox4-7*: 1,335 **(I)**, and 1,370 **(J)** cm^–1^; *r*- and *p*-values for trendlines are reported in Table 8.

One may wonder whether RS can be used for prediction of stalk rot resistance. Currently, resistance determination is achieved by splitting open the stalks and evaluating lesion areas, which is time-consuming and labor-intensive. The smaller the lesion size, the stronger is plant resistance to the pathogen. In this study, we questioned whether RS can be used to predict the lesion size based on changes in the intensities of vibrational bands. We analyzed lesion sizes of four internodes in the field-grown maize at D4, D8, and D12 and changes in intensities of all vibrational bands in the spectra collected from these stalks (Figure 2). We found that lesion size in B73 is correlated with the intensities of three vibrational bands: 986, 1,168, and 1,524 cm^–1^ (Tables 3, 8 and Figure 5). These vibrational bands originate from cellulose (986 cm^–1^), lignin (1,168 cm^–1^), and carotenoids (1,524 cm^–1^).

**TABLE 8:** Vibrational bands that can be used to determine sizes of lesions in the field-grown B73, *lox4-7*, and MP305 with corresponding *r* and *p* values.

**Genotype**	**Vibrational band**	***r***	***p***
B73	986	−0.4073	0.0389
	1168	−0.4392	0.0248
	1524	−0.6052	0.0011
*lox4-7*	1335	−0.412	0.0365
	1370	−0.398	0.044
MP305	522	−0.4164	0.043
	986	−0.4161	0.0431
	1168	−0.4459	0.029
	1601	−0.4294	0.0363
	1630	−0.4351	0.0336

Our results also demonstrate that lesion size in *lox4-7* is correlated with the intensities of the 1,135 cm^–1^ (cellulose) and 1,370 cm^–1^ (aliphatic) bands, whereas lesion size in MP305 is correlated with the intensities of the 522 cm^–1^ (cellulose), 986 cm^–1^ (terpenes), 1,168 cm^–1^ (lignin), 1,601 and 1,630 cm^–1^ bands (phenylpropanoids). These findings demonstrate that progression of stalk rot is associated with a decrease in the content of scaffold molecules (cellulose and lignin), as well as molecules that are directly involved in plant defense (terpenes, phenylpropanoids, and carotenoids). These results also show that RS is a suitable non-invasive and non-destructive alternative to the currently used approach for lesion size determination. Consequently, RS can be used for in-field and greenhouse-based screening for plant resistance to stalk rot.

### Limitations of Raman as a Diagnostic Tool

While the present work has demonstrated the potential of RS as a field diagnostic tool, many open questions remain to be addressed before it can be widely applied. First, it is not yet known how the spectra will vary from location to location when stresses are involved. Our group has demonstrated that potatoes of the same cultivar grown in different locations can be differentiated with high accuracy using PLS-DA (Morey et al., 2020). Additionally, in this study, the precise biochemical origins of the spectral changes observed are not clear. HPLC-MS would be required to identify the associated compounds. Future developments in RS should address these sorts of issues.

Raman spectroscopy also has some practical limitations that make such experiments difficult. First, ambient light must be avoided to acquire good spectra. Scans in the field were acquired at dusk and into the night to avoid ambient light while greenhouse scans were acquired in a dark room attached to the greenhouses. While shrouding with dark plastics or sheets may be feasible, we found that these were inadequate for operation in full light conditions outdoors or in the greenhouse. Instruments with better light exclusion would be ideal for future experiments. Additionally, modern portable instruments often come with built-in software which greatly decrease the rate of scanning. While the acquisition time reported here is 1 s, the actual total duration of a scan was much longer. The CCD camera in the instrument was exposed for 1 s but required steps in the instrument software bloat the total scan time to over 40 s. Devices with leaner software would be ideal for these sorts of analyses.

## Conclusion

Our findings show that RS can be used for highly accurate identification of stalk rot of maize at both early and late stages of disease progression. These diagnostics could be achieved via spectroscopic analysis of both leaves and stalks. In this study, we also investigated the accuracy of disease diagnostics in three varieties of corn grown both in a greenhouse and in field. Our results suggest that accuracy of Raman-based approach is variety-dependent.

On average, the accuracy was higher in the greenhouse compared to the field. We also found that accuracy of correct identification of stalk rot strongly depends on the time point analyzed. It increased from D4 to D8, and from D8 to D12. Since RS diagnostics is based on detection of changes in the plant biochemistry, one can speculate that metabolic changes associated with stalk rot are not linear in maize. Their magnitude increases soon after inoculation which enables highly accurate identification of early stages of stalk rot. However, the magnitude of these changes later decreases. This lowers the accuracy of stalk rot identification at D8. Lastly, the magnitude of biochemical changes increases again at D12, which allows for highly accurate diagnostics of late stages of stalk rot.

Finally, our results show that RS can be used to predict lesion size in stalks that develop after stalk inoculation. Determination of the lesion sizes is used for prediction of maize variety resistance to the pathogen. Our findings show that a spectroscopic analysis of vibrational bands that originate from scaffold molecules such as lignin and cellulose, as well as molecules that are involved in plant signaling and stress response (carotenoids), can be used to predict lesion size in the plant. This discovery demonstrates that RS can be used for digital selection of plant varieties. Considering the portable nature of the spectrometer, non-invasive nature of this innovative spectroscopic approach, and 1 s spectral integration time required for data collection, one can expect that RS will transform conventional approaches used for diagnostics of biotic stresses.

## Data Availability Statement

The raw data supporting the conclusions of this article will be made available by the authors, without undue reservation.

## Author Contributions

CF, LS, and JB: investigation, data curation, and methodology. TD, YA, and DH: investigation. KT: investigation and data curation. MK: methodology, funding acquisition, and supervision. DK: investigation, methodology, funding acquisition, and supervision. All authors contributed to the article and approved the submitted version.

## Conflict of Interest

The authors declare that the research was conducted in the absence of any commercial or financial relationships that could be construed as a potential conflict of interest.

## Publisher’s Note

All claims expressed in this article are solely those of the authors and do not necessarily represent those of their affiliated organizations, or those of the publisher, the editors and the reviewers. Any product that may be evaluated in this article, or claim that may be made by its manufacturer, is not guaranteed or endorsed by the publisher.
